# Supplementary material for: Pre-existing autoimmunity is associated with increased severity of COVID-19: A retrospective cohort study using data from the National COVID Cohort Collaborative (N3C)
Source: medRxiv. 2023 Feb 4:2023.02.02.23285353. Preprint. [Version 1] doi: 10.1101/2023.02.02.23285353 (PMC9915827; doi:10.1101/2023.02.02.23285353)

Supplementary Files

Arjun S. Yadaw[1], Behdad Afzali[2], Nathan Hotaling[1], Hythem Sidky[1], Emily R Pfaff[3], David Sahner[1], Ewy A. Mathé[1]*; "Pre-existing autoimmunity is associated with increased severity of COVID-19: A retrospective cohort study using data from the National COVID Cohort Collaborative (N3C)"

eTable 1. Preexisting Comorbidities evaluated with associated concept set names and IDs.

Preexisting comorbidities were defined as occurring prior to COVID-19 diagnosis.

Pre-existing Comorbidity	Concept Set Names (latest codeset ID)
Myocardial infarction (MI)	Charlson - MI (259495957)
Congestive heart failure (CHF)	Charlson - CHF (359043664)
Peripheral vascular disease (PVD)	Charlson - PVD (376881697)
Stroke	Charlson - Stroke (652711186)
Dementia	Charlson - Dementia (78746470)
Pulmonary Diseases	Charlson - Pulmonary (514953976)
Liver diseases, mild	Charlson - LiverMild (494981955)
Liver diseases, severe	Charlson - LiverSevere (248333963)
Type 2 diabetes mellitus	Type 2 diabetes mellitus (484742674)
Kidney diseases	Charlson - Kidney (220495690)
Cancer	Charlson - Cancer (535274723)
Metastatic cancer (Mets)	Charlson - Mets (378462283)
HIV infection	HIV Infection (382527336)

eTable 2. List of autoimmune diseases with its concept Ids

AID diagnosis cocured prior to the earliest covid diagnosis. AID sorted based on OMOP, Vocabulary Id = SNOMED & Concept Class Id = Clinical Finding from condition_occurence table.

	Autoimmune Disease Names	Concept ID
**Type 1 diabetes**	Type 1 diabetes mellitus with unspecified diabetic retinopathy	1567944
	Type 1 diabetes mellitus with moderate nonproliferative diabetic retinopathy	1567946
	Type 1 diabetes mellitus with oral complications	1567954
	Poorly controlled type 1 diabetes	3194119
	Latent autoimmune diabetes mellitus in adult	4145827

	Type 1 diabetes mellitus with unspecified complications	35206878
	Mild nonproliferative retinopathy due to type 1 diabetes mellitus	37016179
	Hyperglycemia due to type 1 diabetes mellitus	37016348
	Type 1 diabetes mellitus with mild nonproliferative diabetic retinopathy with macular edema, left eye	37200142
	Type 1 diabetes mellitus with mild nonproliferative diabetic retinopathy with macular edema, unspecified eye	37200144
	Type 1 diabetes mellitus with moderate nonproliferative diabetic retinopathy with macular edema, unspecified eye	37200152
	Type 1 diabetes mellitus with severe nonproliferative diabetic retinopathy with macular edema, right eye	37200157
	Type 1 diabetes mellitus with severe nonproliferative diabetic retinopathy with macular edema, unspecified eye	37200160
	Type 1 diabetes mellitus with severe nonproliferative diabetic retinopathy without macular edema, left eye	37200162
	Type 1 diabetes mellitus with proliferative diabetic retinopathy with macular edema, bilateral	37200167
	Type 1 diabetes mellitus with proliferative diabetic retinopathy with traction retinal detachment involving the macula, bilateral	37200172
	Type 1 diabetes mellitus with proliferative diabetic retinopathy with combined traction retinal detachment and rhegmatogenous retinal detachment, left eye	37200181
	Type 1 diabetes mellitus with stable proliferative diabetic retinopathy, bilateral	37200187
	Disorder of eye due to type 1 diabetes mellitus	42538169
	Pre-existing type 1 diabetes mellitus in pregnancy	43531008
	Type 1 diabetes mellitus with hyperglycemia	45533018
	Type 1 diabetes mellitus with other diabetic arthropathy	45537960
	Type 1 diabetes mellitus with unspecified diabetic retinopathy without macular edema	45542736
	Type 1 diabetes mellitus with diabetic peripheral angiopathy without gangrene	45547622
	Type 1 diabetes mellitus with other oral complications	45547623

Type 1 diabetes mellitus with other diabetic ophthalmic complication	45576438
Type 1 diabetes mellitus with mild nonproliferative diabetic retinopathy without macular edema	45591026
Type 1 diabetes mellitus with other diabetic kidney complication	45600637
Type 1 diabetes mellitus with diabetic dermatitis	45600639
Type 1 diabetes mellitus with hypoglycemia without coma	45600640
Hypoglycemic unawareness due to type 1 diabetes mellitus	45757362
Microalbuminuria due to type 1 diabetes mellitus	45757535
Diabetes mellitus type 1 without retinopathy	45757674
Blindness due to type 1 diabetes mellitus	45763585
Neuropathic arthropathy due to type 1 diabetes mellitus	45769830
Severe malnutrition due to type 1 diabetes mellitus	45769833
Hypoglycemia due to type 1 diabetes mellitus	45769876
Chronic kidney disease stage 1 due to type 1 diabetes mellitus	45773576
Diabetes Type 1	45883360
Renal disorder due to type 1 diabetes mellitus	200687
Peripheral circulatory disorder due to type 1 diabetes mellitus	318712
Ketoacidosis due to type 1 diabetes mellitus	439770
Type 1 diabetes mellitus without complication	443412
Type 1 diabetes mellitus maturity onset	4099215
Lumbosacral radiculoplexus neuropathy due to type 1 diabetes mellitus	4143857
Exudative maculopathy due to type 1 diabetes mellitus	4221344
Persistent proteinuria due to type 1 diabetes mellitus	4222553
Ketoacidotic coma due to type 1 diabetes mellitus	4224254
Multiple complications due to type 1 diabetes mellitus	4224709
Cataract due to diabetes mellitus type 1	4225656
Type 1 diabetes mellitus with hypoglycemia without coma	35206877
Moderate nonproliferative retinopathy due to type 1 diabetes mellitus	37016180

	Type 1 diabetes mellitus with mild nonproliferative diabetic retinopathy with macular edema, bilateral	37200143
	Type 1 diabetes mellitus with moderate nonproliferative diabetic retinopathy with macular edema, right eye	37200149
	Type 1 diabetes mellitus with moderate nonproliferative diabetic retinopathy without macular edema, unspecified eye	37200156
	Type 1 diabetes mellitus with proliferative diabetic retinopathy with macular edema, unspecified eye	37200168
	Type 1 diabetes mellitus with proliferative diabetic retinopathy with combined traction retinal detachment and rhegmatogenous retinal detachment, right eye	37200180
	Type 1 diabetes mellitus with proliferative diabetic retinopathy without macular edema, bilateral	37200191
	Type 1 diabetes mellitus with diabetic macular edema, resolved following treatment, unspecified eye	37200197
	Ulcer of heel due to type 1 diabetes mellitus	37312201
	Mixed hyperlipidemia due to type 1 diabetes mellitus	43530660
	Erectile dysfunction due to type 1 diabetes mellitus	43531565
	Type 1 diabetes mellitus, With neurological complications	45533016
	Type 1 diabetes mellitus, With other specified complications	45537959
	Type 1 diabetes mellitus, With ketoacidosis	45542735
	Type 1 diabetes mellitus with other specified complication	45547624
	Type 1 diabetes mellitus with unspecified diabetic retinopathy with macular edema	45552381
	Type 1 diabetes mellitus with diabetic cataract	45552382
	Type 1 diabetes mellitus with hypoglycemia with coma	45552383
	Type 1 diabetes mellitus with moderate nonproliferative diabetic retinopathy with macular edema	45561947
	Type 1 diabetes mellitus with other circulatory complications	45581349
	Type 1 diabetes mellitus with diabetic polyneuropathy	45595795
	Type 1 diabetes mellitus with foot ulcer	45605398
	Retinal edema due to type 1 diabetes mellitus	45757266

Ulcer of foot due to type 1 diabetes mellitus	45757507
Type I diabetes mellitus in remission	45766051
Chronic kidney disease stage 2 due to type 1 diabetes mellitus	45769901
Chronic kidney disease stage 5 due to type 1 diabetes mellitus	45769903
Sensory neuropathy due to type 1 diabetes mellitus	45773567
Chronic kidney disease due to type 1 diabetes mellitus	45773688
Type 1 diabetes mellitus with neurological complications	1567949
Poorly controlled type I diabetes with renal complication	3192052
Poorly controlled type I diabetes with neuropathy	3192955
Type 1 diabetes mellitus with arthropathy	4152858
Persistent microalbuminuria due to type 1 diabetes mellitus	4222687
Gangrene due to type 1 diabetes mellitus	4223303
Mononeuropathy due to type 1 diabetes mellitus	4225055
Retinopathy due to type 1 diabetes mellitus	4227210
Hypoglycemic coma due to type 1 diabetes mellitus	4228112
Type 1 diabetes mellitus without complications	35206879
Type 1 diabetes mellitus with mild nonproliferative diabetic retinopathy with macular edema, right eye	37200141
Type 1 diabetes mellitus with mild nonproliferative diabetic retinopathy without macular edema, right eye	37200145
Type 1 diabetes mellitus with moderate nonproliferative diabetic retinopathy with macular edema, left eye	37200150
Type 1 diabetes mellitus with moderate nonproliferative diabetic retinopathy with macular edema, bilateral	37200151
Type 1 diabetes mellitus with severe nonproliferative diabetic retinopathy with macular edema, bilateral	37200159
Type 1 diabetes mellitus with severe nonproliferative diabetic retinopathy without macular edema, unspecified eye	37200164
Type 1 diabetes mellitus with proliferative diabetic retinopathy with traction retinal detachment involving the macula, right eye	37200170

	Type 1 diabetes mellitus with proliferative diabetic retinopathy with traction retinal detachment involving the macula, left eye	37200171
	Type 1 diabetes mellitus with proliferative diabetic retinopathy with traction retinal detachment not involving the macula, unspecified eye	37200178
	Type 1 diabetes mellitus with stable proliferative diabetic retinopathy, left eye	37200186
	Type 1 diabetes mellitus with diabetic macular edema, resolved following treatment	37200193
	Type 1 diabetes mellitus with diabetic macular edema, resolved following treatment, bilateral	37200196
	Hyperosmolarity due to type 1 diabetes mellitus	42535540
	Type 1 diabetes mellitus with diabetic nephropathy	45552379
	Type 1 diabetes mellitus, With ophthalmic complications	45552380
	Type 1 diabetes mellitus, With unspecified complications	45552384
	Type 1 diabetes mellitus with proliferative diabetic retinopathy without macular edema	45571654
	Type 1 diabetes mellitus	45576436
	Type 1 diabetes mellitus with other skin ulcer	45581350
	Type 1 diabetes mellitus, Without complications	45581351
	Type 1 diabetes mellitus, With renal complications	45586137
	Type 1 diabetes mellitus with other diabetic neurological complication	45586138
	Type 1 diabetes mellitus with diabetic neuropathy, unspecified	45600638
	Type 1 diabetes mellitus, With coma	45755355
	Proteinuria due to type 1 diabetes mellitus	45757604
	Dermopathy due to type 1 diabetes mellitus	45769832
	Chronic kidney disease stage 4 due to type 1 diabetes mellitus	45769902
	Chronic kidney disease stage 3 due to type 1 diabetes mellitus	45771075
	Severe nonproliferative retinopathy due to diabetes mellitus type 1	765373
	Type 1 diabetes mellitus	1567940
	Type 1 diabetes mellitus with mild nonproliferative diabetic retinopathy	1567945
	Type 1 diabetes mellitus with proliferative diabetic retinopathy	1567948

Type 1 diabetes mellitus with other specified complications	1567951
Type 1 diabetes mellitus with diabetic arthropathy	1567952
Type 1 diabetes mellitus with hypoglycemia	1567955
Pre-existing type 1 diabetes mellitus	4063042
Type 1 diabetes mellitus with ulcer	4099214
Small vessel disease due to type 1 diabetes mellitus	4143689
Cranial nerve palsy due to type 1 diabetes mellitus	35626765
Peripheral neuropathy due to type 1 diabetes mellitus	37018566
Type 1 diabetes mellitus with mild nonproliferative diabetic retinopathy without macular edema, left eye	37200146
Type 1 diabetes mellitus with mild nonproliferative diabetic retinopathy without macular edema, bilateral	37200147
Type 1 diabetes mellitus with moderate nonproliferative diabetic retinopathy without macular edema, bilateral	37200155
Type 1 diabetes mellitus with proliferative diabetic retinopathy with traction retinal detachment involving the macula, unspecified eye	37200173
Type 1 diabetes mellitus with proliferative diabetic retinopathy with traction retinal detachment not involving the macula	37200174
Type 1 diabetes mellitus with proliferative diabetic retinopathy with traction retinal detachment not involving the macula, left eye	37200176
Type 1 diabetes mellitus with proliferative diabetic retinopathy with combined traction retinal detachment and rhegmatogenous retinal detachment, unspecified eye	37200183
Type 1 diabetes mellitus with stable proliferative diabetic retinopathy	37200184
Type 1 diabetes mellitus with diabetic macular edema, resolved following treatment, left eye	37200195
Type 1 diabetes mellitus with diabetic autonomic (poly)neuropathy	45533017
Type 1 diabetes mellitus, With multiple complications	45571655
Vitreous hemorrhage due to type 1 diabetes mellitus	45757074
Hyperlipidemia due to type 1 diabetes mellitus	45757432

Proliferative retinopathy due to type 1 diabetes mellitus	45763584
Retinopathy due to unstable diabetes mellitus type 1	45770986
Rubeosis iridis due to type 1 diabetes mellitus	45771533
Skin ulcer of toe due to diabetes mellitus type 1	46269764
Type 1 diabetes mellitus with ophthalmic complications	1567943
Macular edema due to type 1 diabetes mellitus	35626069
Peripheral angiopathy due to type 1 diabetes mellitus	36713094
Acidosis due to type 1 diabetes mellitus	36715571
Dyslipidemia due to type 1 diabetes mellitus	37016353
Polyneuropathy due to type 1 diabetes mellitus	37017431
Type 1 diabetes mellitus with moderate nonproliferative diabetic retinopathy without macular edema, right eye	37200153
Type 1 diabetes mellitus with moderate nonproliferative diabetic retinopathy without macular edema, left eye	37200154
Type 1 diabetes mellitus with severe nonproliferative diabetic retinopathy without macular edema, bilateral	37200163
Type 1 diabetes mellitus with proliferative diabetic retinopathy with macular edema, right eye	37200165
Type 1 diabetes mellitus with proliferative diabetic retinopathy with traction retinal detachment involving the macula	37200169
Type 1 diabetes mellitus with proliferative diabetic retinopathy with combined traction retinal detachment and rhegmatogenous retinal detachment	37200179
Type 1 diabetes mellitus with proliferative diabetic retinopathy without macular edema, unspecified eye	37200192
Type 1 diabetes mellitus with diabetic macular edema, resolved following treatment, right eye	37200194
Neuropathy due to type 1 diabetes mellitus	37312218
Type 1 diabetes mellitus with severe nonproliferative diabetic retinopathy without macular edema	45537958
Type 1 diabetes mellitus with diabetic chronic kidney disease	45547621
Type 1 diabetes mellitus with diabetic amyotrophy	45561948

	Type 1 diabetes mellitus with proliferative diabetic retinopathy with macular edema	45576437
	Type 1 diabetes mellitus with diabetic neuropathic arthropathy	45576440
	Type 1 diabetes mellitus, With peripheral circulatory complications	45595796
	Type 1 diabetes mellitus with ketoacidosis without coma	45600636
	Ischemia of retina due to type 1 diabetes mellitus	45757073
	Nonproliferative diabetic retinopathy due to type 1 diabetes mellitus	45763583
	Osteomyelitis due to type 1 diabetes mellitus	45769834
	Chronic ulcer of skin due to type 1 diabetes mellitus	45769837
	Traction detachment of retina due to type 1 diabetes mellitus	45769873
	Ulcer of forefoot due to type 1 diabetes mellitus	45769892
	End stage renal disease on dialysis due to type 1 diabetes mellitus	45769904
	Type 1 diabetes mellitus	201254
	Disorder of nervous system due to type 1 diabetes mellitus	377821
	Disorder due to type 1 diabetes mellitus	435216
	Hyperosmolality due to uncontrolled type 1 diabetes mellitus	443592
	Type 1 diabetes mellitus with severe nonproliferative diabetic retinopathy	1567947
	Type 1 diabetes mellitus with circulatory complications	1567950
	Poorly controlled type I diabetes with complication	3196797
	Insulin dependent diabetes mellitus type 1A	4047906
	Autonomic neuropathy due to type 1 diabetes mellitus	37016767
	Gastroparesis due to type 1 diabetes mellitus	37017429
	Type 1 diabetes mellitus with mild nonproliferative diabetic retinopathy without macular edema, unspecified eye	37200148
	Type 1 diabetes mellitus with severe nonproliferative diabetic retinopathy with macular edema, left eye	37200158
	Type 1 diabetes mellitus with severe nonproliferative diabetic retinopathy without macular edema, right eye	37200161

	Type 1 diabetes mellitus with proliferative diabetic retinopathy with traction retinal detachment not involving the macula, right eye	37200175
	Type 1 diabetes mellitus with proliferative diabetic retinopathy with traction retinal detachment not involving the macula, bilateral	37200177
	Type 1 diabetes mellitus with diabetic peripheral angiopathy with gangrene	45542737
	Type 1 diabetes mellitus with ketoacidosis with coma	45557110
	Type 1 diabetes mellitus with other skin complications	45566729
	Type 1 diabetes mellitus with mild nonproliferative diabetic retinopathy with macular edema	45595793
	Type 1 diabetes mellitus with moderate nonproliferative diabetic retinopathy without macular edema	45605397
	Hyperosmolar coma due to type 1 diabetes mellitus	201531
	Type 1 diabetes mellitus with ketoacidosis	1567941
	Type 1 diabetes mellitus with kidney complications	1567942
	Type 1 diabetes mellitus with skin complications	1567953
	Poorly controlled type I diabetes with circulatory disorder	3198350
	Insulin dependent diabetes mellitus type 1B	4102018
	Type 1 diabetes mellitus with proliferative diabetic retinopathy with combined traction retinal detachment and rhegmatogenous retinal detachment, bilateral	37200182
	Type 1 diabetes mellitus with stable proliferative diabetic retinopathy, right eye	37200185
	Type 1 diabetes mellitus with stable proliferative diabetic retinopathy, unspecified eye	37200188
	Type 1 diabetes mellitus with proliferative diabetic retinopathy without macular edema, right eye	37200189
	Type 1 diabetes mellitus with proliferative diabetic retinopathy without macular edema, left eye	37200190
	Ulcer of midfoot due to type 1 diabetes mellitus	37312200
	Disorder of nerve co-occurrent and due to type 1 diabetes mellitus	42535539
	Pregnancy and type 1 diabetes mellitus	43531009
	Type 1 diabetes mellitus with periodontal disease	45557111
	Type 1 diabetes mellitus with diabetic mononeuropathy	45576439

	Type 1 diabetes mellitus with severe nonproliferative diabetic retinopathy with macular edema	45595794
	Nephrotic syndrome due to type 1 diabetes mellitus	45769829
	Ankle ulcer due to type 1 diabetes mellitus	45769891
	Ulcer of lower limb due to type 1 diabetes mellitus	45770902
	Hypertension in chronic kidney disease due to type 1 diabetes mellitus	45771067
**Psoriasis**	Circinate and annular pustular psoriasis	4033785
	Plaque psoriasis	4063431
	Psoriasis universalis	4066488
	Juvenile psoriatic arthritis with psoriasis	4079734
	Iritis in psoriatic arthritis	4132495
	Onset of psoriasis in early adulthood (20-40 years)	4270750
	Generalized psoriasis	4292223
	Non-pustular psoriasis of hands and feet	4292227
	Impetigo herpetiformis	133284
	Generalized pustular psoriasis	4031142
	Unstable psoriasis	4031646
	Psoriasis plantaris	4064049
	Psoriasis gyrata	4066487
	Psoriatic arthritis with distal interphalangeal joint involvement	4083682
	Guttate flare of psoriasis with preexisting plaques	4270747
	Late onset psoriasis type 2	4270751
	Chronic large plaque psoriasis	4292222
	Drug-exacerbated psoriasis	4292228
	Psoriasis of scalp margin	4299284
	Acute generalized pustular flare of preexisting plaque psoriasis	4299285
	Onset of psoriasis in adolescence (10-20 years)	4299286
	Erythrodermic psoriasis	4307927
	Pustular psoriasis of palm of hand	37205057
	Psoriatic nail dystrophy	4031140
	Psoriasis of nail	4031649
	Pustular psoriasis in children	4031652
	Psoriasis palmaris	4063433
	Psoriasis annularis	4066830

Juvenile psoriatic arthritis without psoriasis	4083680
Generalized pustular psoriasis, exanthematous type	4103079
Psoriasis of vulva	4223485
Chronic small plaque psoriasis	4270744
Onset of psoriasis in childhood (1-10 years)	4297498
Psoriasis of penis	4299282
X-linked intellectual disability with seizure and psoriasis syndrome	36714528
Psoriasis of anogenital region	36715780
Pustular psoriasis of sole of foot	37205056
Psoriatic arthritis mutilans	46274123
Psoriasis	140168
Arthritis mutilans	4025831
Eczematized psoriasis	4031648
Lapiere type of psoriasis	4033783
Juvenile pustular psoriasis	4033784
Psoriasis inveterata	4066831
Psoriasis punctata	4066832
Chronic guttate pattern psoriasis	4270745
Actively extending plaque psoriasis	4270746
Non-pustular psoriasis of hands	4270748
Onset of psoriasis in infancy (<1 year)	4270749
Guttate psoriasis	4284492
Acute generalized pustular psoriasis de novo	4297497
Familial psoriasis with affected first degree relative	4297499
Psoriasis vulgaris	4307925
Deficiency of interleukin 36 receptor antagonist	37205061
Acute generalized exanthematous pustulosis	45773387
Scalp psoriasis	4031141
Psoriasis-eczema overlap condition	4031647
Rupioid psoriasis	4063432
Psoriasis diffusa	4066485
Juvenile psoriatic arthritis	4079733
Localized pustular psoriasis	4217927
Generalized pustular psoriasis of von Zumbush	4243163
Photoaggravated psoriasis	4292224

	Acute guttate psoriasis	4299281
	Psoriasis with arthropathy	81931
	Infantile pustular psoriasis	4031143
	Flexural psoriasis	4031645
	Psoriatic nail pitting	4031650
	Köbner psoriasis	4031651
	Psoriatic dactylitis	4035742
	Psoriasis circinata	4063430
	Psoriatic arthritis with spine involvement	4064048
	Psoriasis geographica	4066486
	Generalized pustular psoriasis of pregnancy	4080941
	Seborrheic psoriasis	4093619
	Pustular psoriasis of palms and soles	4100184
	Familial psoriasis without affected first degree relative	4270752
	Hypertrophic palmar psoriasis	4292225
	Hypertrophic palmoplantar psoriasis	4292226
	Early onset psoriasis type 1	4292230
	Psoriasis of face	4297496
	Chronic stable plaque psoriasis	4299280
	Psoriasis of perianal skin	4299283
	Familial psoriasis	4299287
	Psoriatic arthritis	40319772
	Acropustulosis of infancy	4033202
	Pustular psoriasis	4063434
	Pustular bacterid	4148213
	Childhood pustular psoriasis	4292229
**Rheumatoid arthritis**	Rheumatoid arthritis	80809
	Seropositive rheumatoid arthritis	4035611
	Rheumatoid arthritis of multiple joints	4117686
	Rheumatoid arthritis - hand joint	4115161
	Rheumatoid arthritis of knee	4116151
	Rheumatoid factor positive rheumatoid arthritis	36684997
	Rheumatoid arthritis of right hand	42534836
	Bilateral rheumatoid arthritis of hands	37209323
	Flare of rheumatoid arthritis	4114444

	Rheumatoid arthritis of right foot	36685023
	Rheumatoid arthritis of bilateral hips	36687003
	Bilateral rheumatoid arthritis of knees	37209322
	Rheumatoid arthritis of left ankle	36685017
**Multiple sclerosis**	Multiple sclerosis	374919
	Relapsing remitting multiple sclerosis	4145049
	Exacerbation of multiple sclerosis	4102337
	Primary progressive multiple sclerosis	4178929
	Secondary progressive multiple sclerosis	4137855
**Lupus**	Systemic lupus erythematosus	257628
	SLE glomerulonephritis syndrome	4285717
	Systemic lupus erythematosus with organ/system involvement	4344158
	Lupus erythematosus	255891
	Systemic lupus erythematosus-related syndrome	4219859
	Cutaneous lupus erythematosus	4324123
	Acute systemic lupus erythematosus	4295179
	Lupus erythematosus overlap syndrome	4291306
	Neonatal lupus erythematosus	4316373
	SLE glomerulonephritis syndrome, WHO class V	4178133
	Discoid lupus erythematosus	4066824
**Autoimmune thyroiditis**	Autoimmune thyroiditis	4281109
	Fibrous autoimmune thyroiditis	138711
	Steroid-responsive encephalopathy associated with autoimmune thyroiditis	36674282
**Idiopathic thrombocytopenic purpura**	Idiopathic thrombocytopenic purpura	4137430
	Chronic idiopathic thrombocytopenic purpura	318397
	Acute idiopathic thrombocytopenic purpura	4102469
	Immune thrombocytopenia	4103532
**Systemic sclerosis**	Systemic sclerosis	134442
	CREST syndrome	4135937
	Lung disease with systemic sclerosis	255304
	Progressive systemic sclerosis	40485046
	Limited systemic sclerosis	4103019
	Sclerodema	4319301

	Systemic sclerosis with limited cutaneous involvement	4185187
**Autoimmune hepatitis**	Autoimmune hepatitis	200762
	Chronic autoimmune hepatitis	36687200
**Autoimmune hemolytic anemia**	Autoimmune hemolytic anemia	441269
	Cold autoimmune hemolytic anemia	4160887
	Drug-induced autoimmune hemolytic anemia	4146936
Ménière's disease	Ménière's disease	79833
	Meniere's disease of left inner ear	36685172
**Polyarteritis nodosa**	Polyarteritis nodosa	320749
	Microscopic polyarteritis nodosa	4344489
	Cutaneous polyarteritis nodosa	4347062
**Inflammatory bowel disease**	Crohn's disease	201606
	Ulcerative colitis	81893
	Inflammatory bowel disease	4074815
**Myasthenia gravis**	Myasthenia gravis	76685
	Myasthenia gravis with exacerbation	43531560
**Others**	Sensorineural hearing loss	374366
	Toxic diffuse goiter	138717
	Sjogren's syndrome	254443
	Raynaud's disease	314962
	Polymyalgia rheumatica	255348
	Megaloblastic anemia due to vitamin B12 deficiency	432588
	Celiac disease	194992
	Antiphospholipid syndrome	4098292
	Aplastic anemia	137829
	Ankylosing spondylitis	437082
	Dermatomyositis	80182
	Chronic inflammatory demyelinating polyradiculoneuropathy	381009
	Primary biliary cholangitis	4135822
	Temporal arteritis	4290976
	Primary sclerosing cholangitis	4058821
	Polymyositis	80800

Lichen planus	132703
Demyelinating disease of central nervous system	375801
Hashimoto thyroiditis	135215
Primary adrenocortical insufficiency	4160059
Alopecia areata	141933
Neuromyelitis optica	380995
Guillain-BarrÈ syndrome	4164770
Behcet's syndrome	436642
Pernicious anemia	432295
Constitutional red cell aplasia and hypoplasia	4146087
Disorder of endocrine ovary	201257
Membranous glomerulonephritis	252365
Graves' disease	4232076
Immunoglobulin A vasculitis	4101602
Stiff-man syndrome	379008
Takayasu's disease	440740
Acute febrile mucocutaneous lymph node syndrome	314381
Pulmonary disease due to allergic granulomatosis angiitis	46273631
Pemphigus vulgaris	4170723
Pustular psoriasis of palms and soles	4100184
Relapsing polychondritis	4216873
Evans syndrome	436956
Systemic onset juvenile chronic arthritis	4116447
Microscopic polyangiitis	44808422
Benign mucous membrane pemphigoid	4142060
Vasculitis of the skin	4182711
Pemphigoid	139899
Pemphigus	135338
Autoimmune disease	434621
Undifferentiated connective tissue disease	4344165
Autonomic neuropathy	4080146
Thromboangiitis obliterans	312939
Nephrotic syndrome, diffuse membranous glomerulonephritis	4058841
Vogt-Koyanagi-Harada disease	4108968
Dermatitis herpetiformis	140487

Giant cell arteritis with polymyalgia rheumatica	4343935
Eaton-Lambert syndrome	4237155
Chronic thyroiditis	137520
Cerebral arteritis	380747
Autoimmune lymphoproliferative syndrome	45765493
Fasciitis with eosinophilia syndrome	4083100
Autoimmune polyendocrinopathy	4223448
Eosinophilic granulomatosis with polyangiitis	4305666
Delayed postmyocardial infarction pericarditis	37311078
Addison's disease	443394
Pemphigus foliaceus	4148690
Thyrotoxic exophthalmos	440108
Progressive external ophthalmoplegia	379027
Autoimmune hypothyroidism	4034815
Endolymphatic hydrops	4321746
Autoimmune encephalitis	4318558
Asteatotic eczema	4308085
Transverse myelopathy syndrome	443904
Psoriasiform dermatitis	4179347
Polymyositis associated with autoimmune disease	4346977
Spongiotic dermatitis	4053289
Pemphigus paraneoplastica	4291435
Exophthalmos due to thyroid eye disease	4214309
Idiopathic transverse myelitis	134330
Autoimmune pancreatitis	40490446
ACTH deficiency	4029439
Balo concentric sclerosis	4046110
Autoimmune encephalitis caused by N-methyl D-aspartate receptor antibody*	764228
Acute inflammatory demyelinating polyneuropathy	43530713
Myasthenic crisis	4215003
Myelofibrosis due to another disorder	760840
Toxic diffuse goiter with exophthalmos	4185522
Pustular eczema	4048079
Granulomatous dermatophytosis	4301243

	Chronic inflammatory demyelinating polyradiculoneuropathy with central nervous system demyelination	4048024
	Rasmussen syndrome*	4041672
	Idiopathic vitiligo	4297816
	Psoriasiform eczema*	4080933
	Secondary hypoparathyroidism	36717646
	Idiopathic membranous glomerulonephritis	36716199
	Erythema nodosum, acute form*	4318267

** These autoimmune diseases have no match at condition occurance table at N3C.*

eTable 3: Univariate and multivariate logistic regression models of severity outcomes unadjusted or adjusted for demographics (January 1, 2020 to June 30, 2022, n = 2,453,799)

	**Life-threatening condition (Yes: 54,932, No: 2,398,867)**				**Hospitalized condition (Yes: 220,353, No: 2,233,446)**			
	**Univariate OR (95% CI)**	***P* value**	**Multivariate adjusting for demographic OR (95% CI)**	***P* value**	**Univariate OR (95% CI)**	***P* value**	**Multivariate adjusted for demographic OR (95% CI)**	***P* value**
**AID/Immunosuppressants(IS) status**								
AID only	2.13 (2.07 - 2.19)	<2.2E-308	1.60 (1.55 - 1.65)	9.50E-189	2.00 (1.96 - 2.03)	<2.2E-308	1.61 (1.58 - 1.63)	<2.2E-308
IS only	2.65 (2.59 - 2.71)	<2.2E-308	1.97 (1.92 - 2.01)	<2.2E-308	2.20 (2.17 - 2.23)	<2.2E-308	1.65 (1.63 - 1.67)	<2.2E-308
AID + IS	3.75 (3.62 - 3.89)	<2.2E-308	2.47 (2.38 - 2.56)	<2.2E-308	3.18 (3.11 - 3.24)	<2.2E-308	2.17 (2.12 - 2.21)	<2.2E-308
**Demographics**								
**Age**	NA		3.79 (3.75 - 3.83)	<2.2E-308	NA		2.46 (2.44 - 2.47)	<2.2E-308
**BMI**	NA		1.14 (1.13 - 1.15)	2.38E-235	NA		1.16 (1.15 - 1.17)	<2.2E-308
**Gender**								
Female	NA		1 [Reference]		NA		1 [Reference]	
Male	NA		1.66 (1.63 - 1.69)	<2.2E-308	NA		1.38 (1.37 - 1.39)	<2.2E-308
**Race**								
White	NA		1 [Reference]		NA		1 [Reference]	
Others/unknown	NA		1.15 (1.11 - 1.19)	1.01E-15	NA		1.16 (1.14 - 1.18)	7.59E-60
Black or African American	NA		1.63 (1.59 - 1.67)	<2.2E-308	NA		2.03 (2.01 - 2.06)	<2.2E-308
Race asian	NA		1.40 (1.32 - 1.49)	3.92E-28	NA		1.43 (1.39 - 1.47)	5.50E-113
**Ethnicity**								
Not Hispanic or Latino	NA		1 [Reference]		NA		1 [Reference]	
Hispanic or Latino	NA		1.28 (1.23 - 1.32)	4.08E-42	NA		1.46 (1.44 - 1.49)	<2.2E-308
**Smoking status**								
Nonsmoker	NA		1 [Reference]		NA		1 [Reference]	
Current or Former	NA		1.67 (1.63 - 1.71)	<2.2E-308	NA		1.86 (1.84 - 1.89)	<2.2E-308

Demographic factors include age, BMI, gender, race, ethnicity, smoking status.

eTable 4. Logistic regression models of AID, IS and AID-IS association with COVID-19 severity (hospitalization or life-threatening), stratified by race

	Race Black				Race White			
	Life-threatening condition; (Yes: 9,410, No: 333,101) OR (95% CI)	*P* value	Hospitalized condition; (Yes: 45,426, No: 297,085) OR (95% CI)	*P* value	Life-threatening condition; (Yes: 39,162, No: 1,751,319) OR (95% CI)	*P* value	Hospitalized condition; (Yes: 159,758, No: 1,789,162) OR (95% CI)	*P* value
**AID/Immunosuppressants(IS) status**								
AID only	1.16 (1.06 - 1.26)	6.92E-04	1.26(1.21 - 1.32)	6.37E-26	1.12(1.08 - 1.16)	2.34E-09	1.19(1.17 - 1.22)	7.10E-61
IS only	1.25 (1.18 - 1.33)	3.24E-14	1.13(1.10 - 1.17)	7.53E-15	1.27(1.23 - 1.31)	5.50E-54	1.20(1.18 - 1.22)	1.53E-87
AID + IS	1.39 (1.27 - 1.52)	3.60E-13	1.31(1.24 - 1.38)	1.27E-23	1.33(1.27 - 1.39)	1.04E-31	1.29(1.25 - 1.33)	6.19E-65
**Demographics**								
**Age**	2.28 (2.21 - 2.34)	<2.2E-308	1.65(1.63 - 1.67)	<2.2E-308	3.07(3.02 - 3.11)	<2.2E-308	2.09(2.07 - 2.10)	<2.2E-308
**BMI**	1.05 (1.03 - 1.07)	3.00E-09	1.08(1.07 - 1.09)	4.72E-73	1.09(1.08 - 1.10)	8.26E-57	1.11(1.11 - 1.12)	<2.2E-308
**Gender**								
Female	1 [Reference]		1 [Reference]		1 [Reference]		1 [Reference]	
Male	1.51(1.44 - 1.57)	2.69E-71	1.29(1.27-1.32)	3.41E-114	1.48(1.45 - 1.51)	6.75E-279	1.31(1.29 - 1.32)	<2.2E-308
**Ethnicity**								
Not Hispanic or Latino	1 [Reference]		1 [Reference]		1 [Reference]		1 [Reference]	
Hispanic or Latino	0.60(0.49 - 0.73)	5.86E-07	0.63(0.58-0.69)	8.88E-28	1.09(1.04 - 1.15)	5.03E-04	1.39(1.36 - 1.42)	2.83E-174
**Smoking status**								
Nonsmoker	1 [Reference]		1 [Reference]		1 [Reference]		1 [Reference]	
Current or Former	1.25(1.19 - 1.32)	5.66E-19	1.23(1.19-1.26)	6.35E-53	1.38(1.34 - 1.42)	9.50E-104	1.80(1.77 - 1.83)	<2.2E-308
**Preexisting Comorbidities**								
Cardiovascular disease	1.95(1.85 - 2.05)	1.46E-140	1.75(1.70-1.80)	<2.2E-308	1.63(1.59 - 1.67)	<2.2E-308	1.64(1.62 - 1.67)	<2.2E-308
Dementia	2.10(1.94 - 2.27)	3.65E-75	1.85(1.74-1.97)	3.15E-84	2.31(2.23 - 2.40)	<2.2E-308	2.00(1.95 - 2.06)	<2.2E-308
Chronic pulmonary disease	1.13(1.07 - 1.19)	2.55E-06	1.22(1.18-1.25)	2.22E-48	1.28(1.25 - 1.32)	1.24E-83	1.33(1.31 - 1.35)	<2.2E-308
Liver mild	1.36(1.27 - 1.45)	1.56E-18	1.25(1.20-1.31)	1.13E-27	1.35(1.30 - 1.40)	3.05E-54	1.27(1.24 - 1.30)	5.47E-96
Liver severe	2.59(2.26 - 2.96)	1.34E-43	1.88(1.70-2.09)	1.02E-32	3.14(2.94 - 3.35)	6.16E-253	2.36(2.25 - 2.48)	7.27E-260
Kidney disease	1.93(1.84 - 2.04)	1.56E-137	2.06(2.00-2.13)	<2.2E-308	1.70(1.65 - 1.74)	<2.2E-308	1.78(1.75 - 1.82)	<2.2E-308
Cancer	1.31(1.22 - 1.40)	1.39E-15	1.14(1.09-1.19)	5.32E-10	1.45(1.41 - 1.50)	5.47E-133	1.24(1.21 - 1.26)	8.22E-104
Metastatic cancer	3.29(2.94 - 3.67)	8.00E-99	2.12(1.95-2.31)	5.29E-67	3.36(3.19 - 3.53)	<2.2E-308	2.17(2.09 - 2.26)	<2.2E-308
Type 2 Diabetes Mellitus	1.31(1.25 - 1.37)	1.65E-27	1.41(1.37-1.44)	2.98E-152	1.32(1.29 - 1.36)	2.85E-107	1.48(1.46 - 1.50)	<2.2E-308
HIV	1.16(1.00 - 1.34)	4.98E-02	1.34(1.24-1.44)	1.77E-14	1.23(1.02 - 1.48)	2.70E-02	1.27(1.15 - 1.40)	1.45554E-06

Demographic factors include age, BMI, gender, race, ethnicity, smoking status.
Comorbidities include cardiovascular disease (MI + CHF + PVD + stroke), dementia, pulmonary disease, liver disease (mild and severe), Type 2 diabetes, kidney disease, cancer (metastatic and non-metastatic), and HIV infection.

eTable 5. Logistic regression models of AID, IS and AID-IS association with COVID-19 severity (hospitalization or life-threatening), stratified by gender

	Gender Male				Gender Female			
	Life-threatening condition; (Yes: 29,476, No: 969,650) OR (95% CI)	*P* value	Hospitalized condition; (Yes: 106,365, No: 892,761) OR (95% CI)	*P* value	Life-threatening condition; (Yes: 25,456, No: 1,429,217) OR (95% CI)	*P* value	Hospitalized condition; (Yes: 113,988, No: 1,340,685) OR (95% CI)	*P* value
**AID/Immunosuppressants(IS) status**								
AID only	1.10(1.04 - 1.15)	2.63E-04	1.21(1.17 - 1.24)	9.17E-38	1.16(1.11 - 1.21)	5.66E-11	1.21(1.19 - 1.24)	3.91E-61
IS only	1.26(1.22 - 1.31)	4.65E-40	1.23(1.20 - 1.25)	2.35E-82	1.27(1.22 - 1.32)	2.82E-36	1.15(1.13 - 1.18)	1.44E-44
AID + IS	1.33(1.26 - 1.42)	6.04E-21	1.34(1.29 - 1.39)	3.24E-49	1.34(1.27 - 1.41)	5.19E-27	1.29(1.25 - 1.33)	2.37E-60
**Demographics**								
**Age**	2.70(2.66 - 2.75)	<2.2E-308	2.07(2.06 - 2.09)	<2.2E-308	2.98(2.93 - 3.03)	<2.2E-308	1.84(1.83 - 1.85)	<2.2E-308
**BMI**	1.04(1.03 - 1.05)	2.68E-09	1.07(1.07 - 1.08)	1.18E-81	1.11(1.09 - 1.12)	5.79E-75	1.11(1.11 - 1.12)	<2.2E-308
**Race**								
White	1 [Reference]		1 [Reference]		1 [Reference]		1 [Reference]	
Others/unknown	1.21(1.16 - 1.27)	7.55E-16	1.13(1.10 - 1.16)	2.87E-19	1.18(1.11 - 1.24)	3.72E-09	1.23(1.20 - 1.26)	2.16E-63
Black or African American	1.40(1.35 - 1.45)	2.72E-76	1.81(1.78 - 1.85)	<2.2E-308	1.30(1.26 - 1.35)	7.20E-49	1.74(1.71 - 1.77)	<2.2E-308
Race asian	1.27(1.16 - 1.38)	3.23E-08	1.27(1.21 - 1.33)	1.98E-23	1.36(1.24 - 1.49)	2.73E-11	1.43(1.37 - 1.49)	2.36E-59
**Ethnicity**								
Not Hispanic or Latino	1 [Reference]		1 [Reference]		1 [Reference]		1 [Reference]	
Hispanic or Latino	1.29(1.23 - 1.36)	3.11E-25	1.42(1.38 - 1.46)	1.94E-158	1.05(0.99 - 1.11)	8.88E-02	1.36(1.32 - 1.39)	6.31E-146
**Smoking status**								
Nonsmoker	1 [Reference]		1 [Reference]		1 [Reference]		1 [Reference]	
Current or Former	1.34(1.30 - 1.38)	1.11E-74	1.66(1.63 - 1.69)		1.41(1.36 - 1.46)	2.37E-84	1.55(1.53 - 1.58)	<2.2E-308
**Preexisting Comorbidities**								
Cardiovascular disease	1.63(1.58 - 1.68)	2.13E-237	1.59(1.57 - 1.62)	<2.2E-308	1.77(1.72 - 1.83)	8.72E-283	1.75(1.72 - 1.78)	<2.2E-308
Dementia	2.21(2.10 - 2.31)	2.11E-239	1.90(1.83 - 1.97)	1.98E-229	2.21(2.12 - 2.31)	2.27E-278	2.09(2.02 - 2.16)	<2.2E-308
Chronic pulmonary disease	1.23(1.19 - 1.27)	5.63E-41	1.28(1.25 - 1.30)	3.26E-148	1.23(1.19 - 1.27)	1.03E-40	1.29(1.27 - 1.31)	2.54E-209
Liver mild	1.34(1.29 - 1.40)	1.54E-42	1.32(1.29 - 1.36)	2.99E-96	1.34(1.28 - 1.40)	2.52E-37	1.18(1.15 - 1.21)	3.42E-38
Liver severe	2.80(2.60 - 3.01)	1.80E-169	2.23(2.11 - 2.35)	7.48E-178	3.43(3.15 - 3.72)	2.73E-186	2.45(2.31 - 2.61)	1.16E-181
Kidney disease	1.71(1.66 - 1.76)	1.68E-253	1.83(1.80 - 1.87)	<2.2E-308	1.86(1.80 - 1.92)	2.22E-291	1.94(1.90 - 1.98)	<2.2E-308
Cancer	1.40(1.35 - 1.45)	2.28E-80	1.18(1.15 - 1.21)	1.01E-43	1.54(1.48 - 1.60)	1.44E-104	1.27(1.24 - 1.30)	2.10E-88
Metastatic cancer	2.86(2.69 - 3.03)	5.54E-262	2.06(1.96 - 2.16)	5.11E-189	3.91(3.67 - 4.17)	<2.2E-308	2.33(2.22 - 2.44)	2.75E-271
Type 2 Diabetes Mellitus	1.30(1.26 - 1.33)	6.33E-73	1.45(1.42 - 1.47)	<2.2E-308	1.35(1.31 - 1.39)	3.05E-81	1.48(1.46 - 1.50)	<2.2E-308
HIV	1.16(1.02 - 1.32)	2.15E-02	1.19(1.11 - 1.27)	1.59E-06	1.37(1.13 - 1.65)	1.20E-03	1.41(1.29 - 1.55	3.59E-13

Demographic factors include age, BMI, gender, race, ethnicity, smoking status.
Comorbidities include cardiovascular disease (MI + CHF + PVD + stroke), dementia, pulmonary disease, liver disease (mild and severe), Type 2 diabetes, kidney disease, cancer (metastatic and non-metastatic), and HIV infection.

eTable 6. Multivariate logistic regression model of severity outcomes adjusted for demographics, preexisting comorbidities, and COVID-19 prevention/intervention vaccination status and antiviral treatment (December 23, 2021 to June 30, 2022, n = 248,743)

	Life-threatening condition; (Yes: 2,581, No: 246,162) OR (95% CI)	*P* value	Hospitalized condition; (Yes: 13,431, No: 235,312) OR (95% CI)	*P* value
**AID/Immunosuppressants(IS) status**				
AID only	1.18(1.02 - 1.36)	2.49E-02	1.34(1.25 - 1.43)	8.60E-18
IS only	1.60(1.42 - 1.81)	4.71E-14	1.61(1.51 - 1.72)	2.08E-50
AID + IS	1.94(1.63 - 2.30)	1.35E-13	1.90(1.73 - 2.10)	2.39E-39
**COVID-19 prevention/intervention**				
Vaccination status	0.72(0.70 - 0.75)	4.52E-76	0.69(0.68 - 0.70)	<2.2E-308
Antiviral treatment	0.31(0.21 - 0.45)	4.26E-10	0.30(0.25 - 0.36)	1.39E-40
**Age**	3.03(2.86 - 3.20)	<2.2E-308	1.95(1.91 - 2.00)	<2.2E-308
**BMI**	1.03(1.00 - 1.07)	6.18E-02	1.00(0.99 - 1.02)	9.99E-01
**Gender**				
Female	1 [Reference]		1 [Reference]	
Male	1.60(1.48 - 1.74)	1.16E-28	1.27(1.22 - 1.31)	3.65E-33
**Race**				
White	1 [Reference]		1 [Reference]	
Others/unknown	0.93(0.76 - 1.13)	4.61E-01	0.96(0.89 - 1.05)	3.96E-01
Black or African American	1.10(0.98 - 1.24)	1.10E-01	1.45(1.38 - 1.52)	3.46E-47
Race asian	0.93(0.67 - 1.29)	6.71E-01	0.76(0.65 - 0.89)	4.91E-04
**Ethnicity**				
Not Hispanic or Latino	1 [Reference]		1 [Reference]	
Hispanic or Latino	1.01(0.81 - 1.24)	9.61E-01	1.56(1.44 - 1.69)	1.53E-27
**Smoking status**				
Nonsmoker	1 [Reference]		1 [Reference]	
Current or Former	1.95(1.76 - 2.17)	1.10E-35	2.03(1.93 - 2.13)	1.86E-166
**Preexisting Comorbidities**				
Cardiovascular disease	1.82(1.65 - 2.00)	8.95E-34	1.90(1.81 - 1.99)	2.19E-156
Dementia	1.98(1.72 - 2.29)	1.84E-20	1.86(1.69 - 2.05)	1.26E-35
Chronic pulmonary disease	1.37(1.25 - 1.50)	3.65E-11	1.41(1.35 - 1.48)	5.08E-50
Liver mild	1.41(1.23 - 1.61)	6.23E-07	1.23(1.15 - 1.32)	5.43E-09
Liver severe	3.17(2.53 - 3.97)	6.38E-24	2.53(2.18 - 2.93)	1.48E-34
Kidney disease	1.75(1.58 - 1.94)	1.45E-27	2.08(1.97 -2.19)	1.89E-153
Cancer	1.49(1.33 - 1.67)	8.20E-12	1.28(1.20 - 1.37)	3.71E-14
Metastatic cancer	3.80(3.20 - 4.52)	1.42E-51	2.29(2.03 - 2.59)	2.19E-40
Type 2 Diabetes Mellitus	1.29(1.18 - 1.42)	6.66E-08	1.46(1.40 - 1.53)	3.99E-59
HIV	0.68(0.33 - 1.40)	2.94E-01	1.36(1.06 - 1.73)	1.42E-02

eFigure 1. Definition of patients with AID or exposed to immunosuppressants prior to COVID-19 diagnosis. A. COVID-19 patients were dichotomized based on whether or not they were diagnosed with AID Prior to their COVID-19 diagnosis date. **B.** COVID-19 patients were dichotomized based on whether or not they were exposed to immunosuppressants drugs prior to COVID-19 diagnosis date. **C.** Cohort created for vaccinated and antiviral treatment individuals between 12/23/2021 to 06/30/2022.

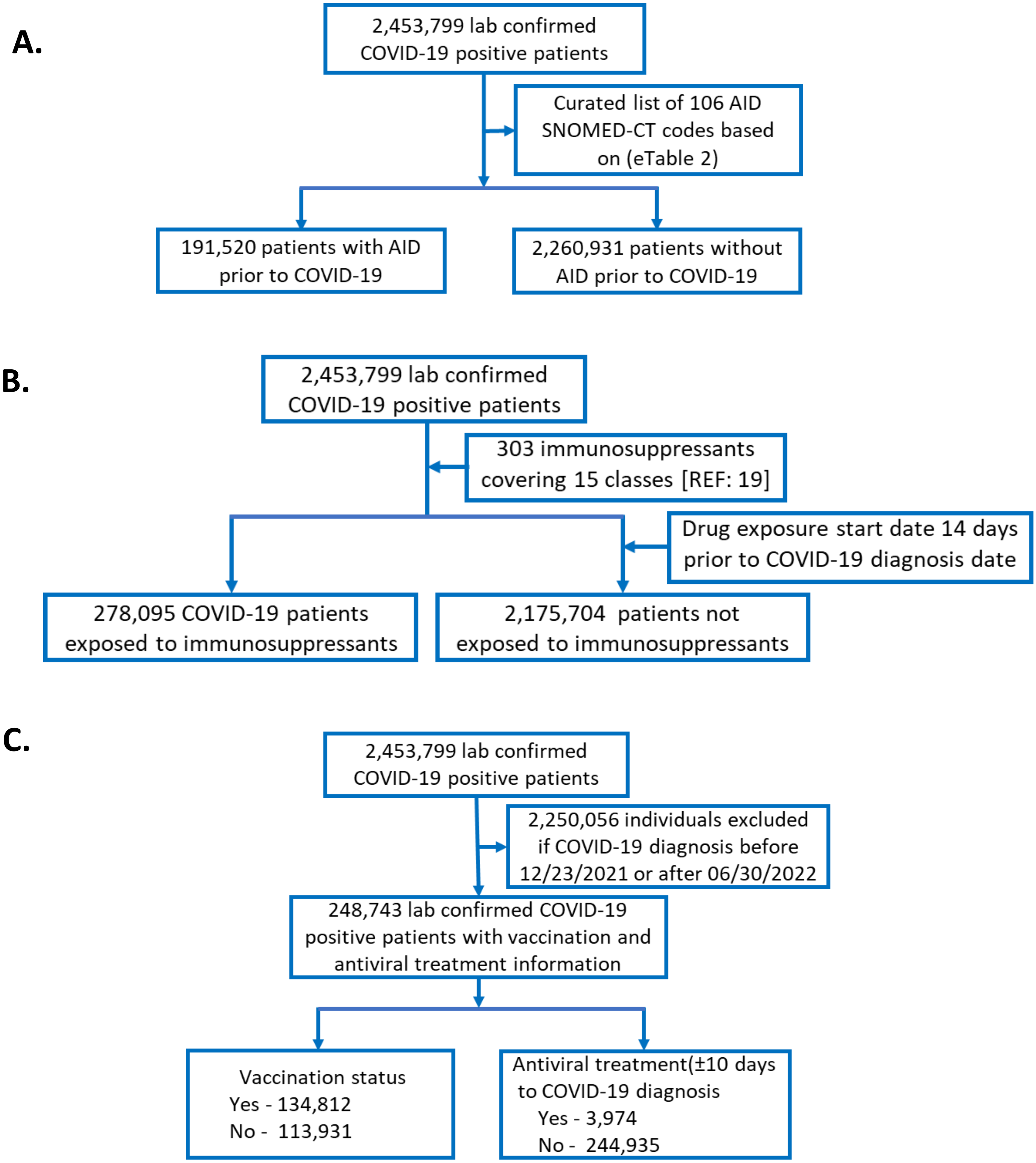

eFigure 2: Statistical models evaluated in COVID-19 patients within N3C to evaluate associations of preexisting AID and/or exposure to IS with COVID-19 severity outcomes. Severity outcomes are defined as hospitalization (Yes/No) or life-threatening disease (Yes/No). Demographic covariables include one each for age, BMI, gender, race, ethnicity, smoking status. Preexisting comorbidities include myocardial infarction (MI), congestive heart failure (CHF), peripheral vascular disease (PVD), stroke, dementia, pulmonary diseases, liver disease (mild and severe), type-2 diabetes mellitus, kidney disease, cancer (metastatic and non-metastatic), and human immunodeficiency virus (HIV). AID represents patients diagnosed with a preexisting AID only, IS represents patients with a preexisting exposure to IS only, and AID/IS represents patients with a preexisting AID diagnosis and exposure to IS.

Models adjusted for demographics and preexisting comorbidities (Table 2 & eTable3)

Severity ~ AID + IS + AID/IS + demographic + preexisting comorbidities

Association between TNF inhibitors and COVID-19 severity outcomes in AID patients (Table3)

Severity ~ TNF inhibitors + other IS* + demographic + preexisting comorbidities

- all other IS individually as defined in methods

Models adjusted for demographics and preexisting comorbidities and stratified by race and gender (eTable4 & eTable5)

Severity ~ AID + IS + AID/IS + demographic + preexisting comorbidities

***Models adjusted for demographics, preexisting comorbidities,* and COVID-19 prevention/intervention (*eTable6*)**

Severity ~ AID + IS + selected antiviral treatment + vaccination status + demographic + preexisting comorbidities

eFigure3: Severity outcome distribution by AID and immunosuppressant exposures. Percentage of COVID-19 patients with or without life-threatening disease and with or without hospitalization for the top 20 AIDs (A, C) and top 15 immunosuppressants (B, D). Patients are considered having a life-threatening disease if they are classified as ”Dead” or “Severe”.

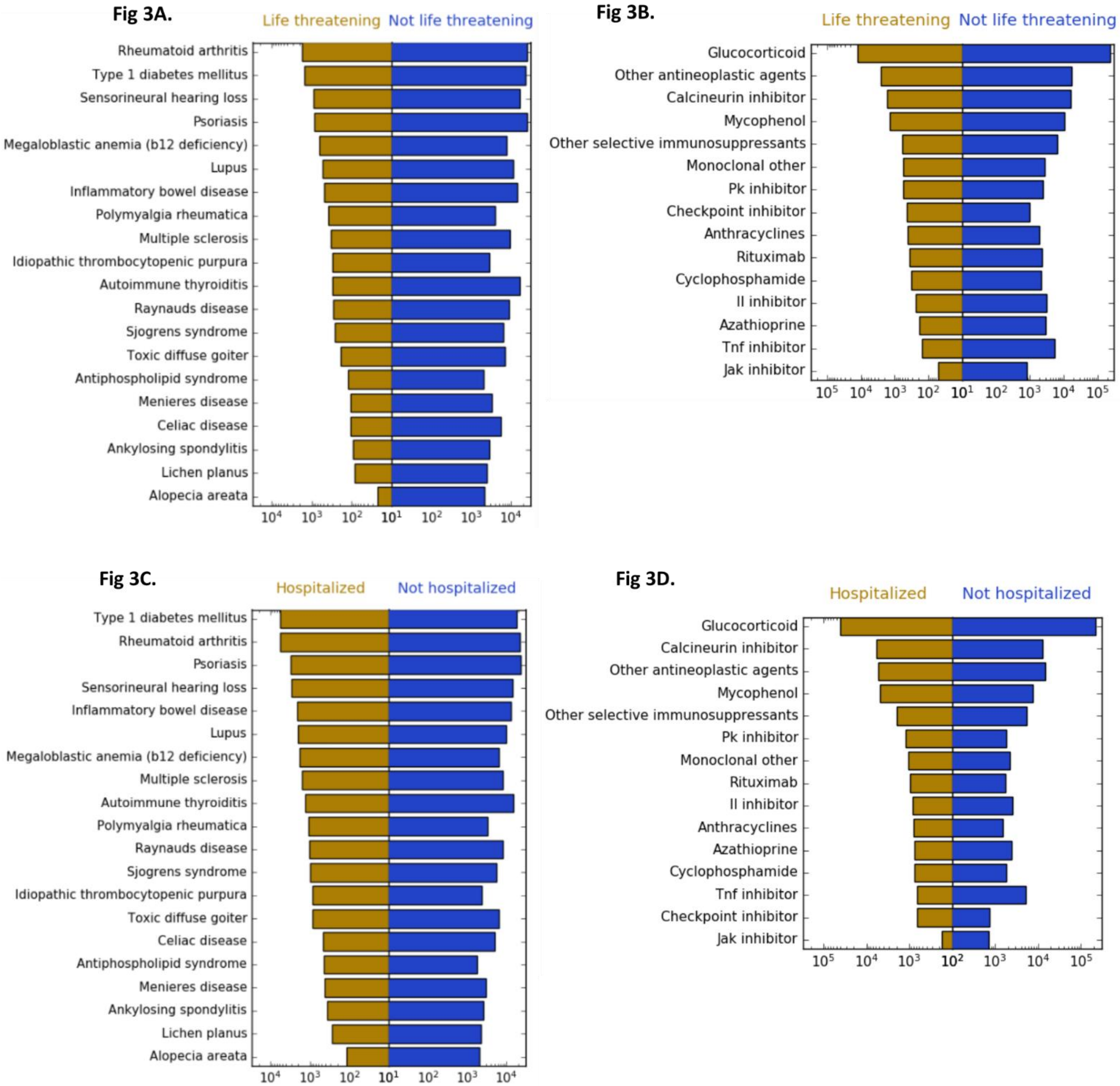

Supplement: Supplement 1 [file media-1.pdf]
